# Parental Confidence in Relation to Antipyretic Use, Warning Signs, Symptoms and Well-Being in Fever Management—Results from an App-Based Registry

**DOI:** 10.3390/ijerph192114502

**Published:** 2022-11-04

**Authors:** Ricarda Möhler, Ekkehart Jenetzky, Silke Schwarz, Moritz Gwiasda, Larisa Rathjens, Henrik Szoke, David Martin

**Affiliations:** 1 Faculty of Health, School of Medicine, Witten/Herdecke University, 58448 Witten, Germany; 2 Department of Child and Adolescent Psychiatry and Psychotherapy, University Medical Center of the Johannes-Gutenberg-University Mainz, 55131 Mainz, Germany; 3 Department of Integrative Medicine, Faculty of Health Sciences, University of Pécs, 7622 Pecs, Hungary; 4 Department of Pediatrics, Eberhard-Karls University Tübingen, 72076 Tübingen, Germany

**Keywords:** parent–child relationship, fever of unknown origin, caregiver, antipyretics, ecological momentary assessment, children’s health

## Abstract

Parents’ confidence regarding their children’s fever is a key factor in its management and there is still unnecessary anxiety and associated antipyretic overuse. The FeverApp application collects naturalistic real-time data on febrile infections and educates parents on fever management. Logistic regression examined the associations between (1) parental confidence and (2) antipyretics use with fever relevant parameters. First entry data of 3721 children (mean age 21 months; SD 22.97) was assessed. A total of 58.0% of parents felt confident upon first fever documentation. Warning signs [OR = 0.49, 95% CI: 0.40–0.61], dehydration [OR = 0.65, 95% CI: 0.52–0.81], fever [OR = 0.67, 95% CI: 0.57–0.80] and having a female child [OR = 0.77, 95% CI: 0.66–0.90] had the highest negative association with parental confidence. Antipyretics were used initially in 14.7% of children. Fever had the highest positive [OR = 2.58, 95% CI: 1.89–3.50] and well-being the highest negative association with antipyretic use [OR = 0.37, 95% CI: 0.22–0.63). In the first entry data, parental confidence was related to children’s health condition in a reasonable medical manner. The use of antipyretics was mostly associated with febrile temperature, but also low well-being. Thus, associations were partly in accordance with recent guidelines.

## 1. Introduction

Parents play an important role in the treatment of fever in children. They treat them at home and bring them to the doctor’s office or hospital for further diagnosis and treatment. Since it is recommended to contact the physician if one feels unsafe in treating his or her child’s fever [1] and when parents feel safe, fewer antipyretics and antibiotics should be administered and fewer health services used [2]. Thus, parents’ confidence is a very important factor in the management of their children’s fever.

In 1980, B.D. Schmitt examined parents who brought their children to a hospital-based pediatric clinic [3]. In his study, parents showed an over-concern regarding fever, which he called “fever phobia”. Parents believed that fever, even at low temperatures, could cause serious neurological problems; therefore, they treated fever aggressively with antipyretics [3]. Over the past 40 years, several other studies taking place in the USA, UK, Canada, Saudi Arabia, Turkey and Germany tried to examine the parental perception of fever and the fears they face [4,5,6,7,8,9,10,11,12]. These fears often relate to brain damage, convulsions, seizures, dehydration and even death [13]. Recent studies show that the reasons for fever reduction should be mainly to calm the child in discomfort [14,15]. However, to increase the feeling of their own safety and to avoid possible harm, parents try to reduce fever [12] and antipyretics are used more frequently than recommended [6,16], even at low temperatures [3,4,6,8]. The use of antipyretics is not necessarily helpful because fever can be beneficial in reducing bacteria and virus load in infectious diseases and mortality in severe cases [15]. Seizures are not prevented [14,15] and antipyretics at high and frequent dosing can even be poisoning [17].

Previous studies examined parental anxiety and antipyretic use and its correlates mainly in hospitals or emergency departments [3,5,6,7,8,10,18], in schools [7] or kindergartens [12]. The used methods where (retrospective) questionnaires [3,4,5,6,7,8,9,11,12], asking about fever knowledge and their worries and behaviors regarding fever management.

Parents in emergency departments or hospital might be a more vulnerable group and show more anxiety than other parents. Questionnaires represent the attitudes of parents but not the actual behavior in such a situation. Therefore, it would be an addition to the recent research if data from fever management at home with the actual feelings of parents and their behavior was available.

The analyzed data from this study comes from the FeverApp application, which is an ecological momentary assessment (EMA) of fever management in families and may represent naturalistic associations within an acute febrile episode. Within the app, parents can document children’s fever episodes and inform themselves about fever management. Parental confidence is defined in the app as “having the confidence to deal with the fever of ones child appropriately”. This is not a direct question about anxiety regarding a child’s fever, but represents a continuum between fear and confidence and a more salutogenetic approach. This approach was also chosen in another study [12].

Therefore, the aim of this study was to specify the influences of parental confidence in the beginning of a febrile episode of their child by examining the associations between parental confidence with sociodemographic and acute fever parameters. Second, we investigated whether parental confidence has an association with the administration of antipyretics in relation to other fever-relevant and sociodemographic parameters.

The aim was to find out whether parental confidence and antipyretic use are associated with the actual condition of the children or whether there is an underlying concern that should be explained and improved. The ecological momentary assessment allows us to observe the actual behavior at home.

## 2. Methods

### 2.1. Study Design

The data used in this study was collected in the German FeverApp Registry, which is an EMA of fever management in families. It was first introduced in 2019 and recorded over 12,388 fever episodes from 6108 children by the end of January 2022. The main aim of FeverApp is to assess guideline adherence regarding fever management in children [1]. The data collected is stored on a university server and can be used for several scientific questions. Within this study, a cross-sectional examination of the first entry of every child of every family was conducted to get an observational overview of the relationship between parental confidence and antipyretics use with several fever relevant parameters at the beginning of a fever episode.

The FeverApp can be downloaded for free in app stores, but for registration, families need a code, which they get from participating pediatricians. This was designed to increase scientific controllability and transparency. Pediatricians can register themselves on the Website of the FeverApp (https://www.feverapp.de/, accessed on 16 September 2022), but it is also possible for parents to register themselves on the website if they want to use the app but their pediatrician does not want to participate.

The family is the main observation object, which can be distinguished between caregivers in different documenting roles (mother, father, grandmother, grandfather, etc.). Observed children can be entered as different profiles if there is more than one child. For each child, the time points of a fever episode, as well as multiple fever episodes, may be documented. Parents can use the FeverApp anytime their child has a fever and they feel the need to use it.

**Figure 1:**
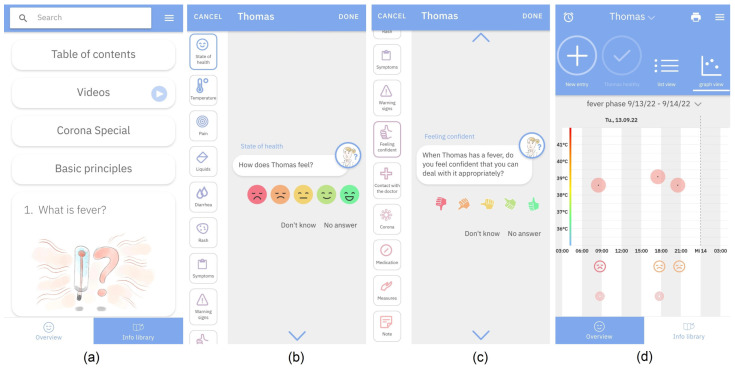
FeverApp view, (**a**) Info-Library; (**b**) Documentation-Tool with well-being as an example (**c**) Documentation-Tool with parental-confidence as an example; (**d**) Graph view of entries.

The app functions as a documentation and information tool. In the initial entry, it collects sociodemographic parameters such as caregiver’s age, nationality, school degree and children’s age and gender. The anamnestic parameters of children are also queried in the initial collection, such as amount and duration of previous fever episodes, as well as chronic diseases. Acute fever-related parameters during a fever episode, such as vaccinations or corona infections, temperature, well-being, symptoms, warning signs, measures, medication intake and current parental confidence in the management of their children’s fever are continuously questioned in every entry (Figure 1b,c).

After every entry, caregivers receive a short summary of the symptoms and get advice about which chapter of the Info-Library they can visit. After several entries, they get an overview about the progress (Figure 1d). The Info-Library consists of 23 chapters regarding fever and its management, as well as educational videos, specific information for corona and basic principles, such as when to go to a doctor or when medication is not necessary (Figure 1a). These case report forms were validated with a pediatricians office regarding the evaluation of data completeness and concordance within the FeverApp [19].

### 2.2. Sample Definition

**Figure 2:**
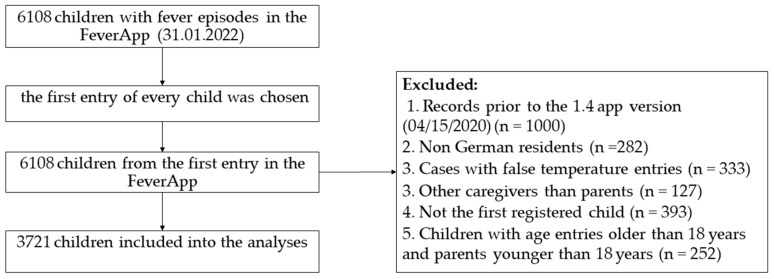
Flowchart describing the selection of included children in the analyses.

The selected sample corresponded to the first entry of the first episode of one child in each family. To date (31 January 2022), 6108 children were registered in the FeverApp. Records prior to the 1.4 app version (15 April 2020) were filtered because in App-Version 1.3., medication, well-being and symptoms such as warning signs and dehydration were collected separately. In this analysis, only data from German residents were considered. To keep confidence clear and interpretable, caregivers were also filtered and only parents were included in the analyses. To avoid bias from multiple parent data, only the first registered child per parent was included in the analyses. Adult profiles and underage parents were excluded. Finally, 3721 children and their caregivers who entered the first entry of the first episode were analyzed (Figure 2).

### 2.3. Variable Selection

The variable selection was based on theoretical deliberations. Parents’ age, their migration background and their school degree showed to have an impact on parental confidence regarding children’s fever management in some studies [11,12,20], so they were included into the analyses. Children’s age, gender and a previous febrile episode in the last 12 months were also included. Age affects the threshold and treatment of fever since fever is diagnosed from a lower degree in younger children and parents should seek help from a doctor earlier when their child is younger [14]. A past febrile episode may be an indicator of experience with fever, and experience with a febrile child potentially affects parental confidence [21]. Relevant current fever parameters such as temperature, well-being, warning signs, symptoms and pain in parental view were selected because they are key factors in fever management of parents because choosing when to go to a doctor or get a treatment depends on these factors [14,15]. Finally, parents’ current confidence in the management of their children’s fever and the current use of antipyretics in the management of their children’s fever were included as dependent variables.

### 2.4. Variable Description

Parent’s school education was dichotomized as “general qualification for university entrance” and “no school degree to advanced technical college entrance qualification” based on the median. Past fever episodes were also dichotomized as “no fever episode in the past year” and “at least one fever episode in the past year” based on the median.

Child temperature was dichotomized as “no-fever” and “fever” or “febrile temperature” according to the threshold of fever in children ≥ 38.5° Celsius [14]. Children’s well-being, based on a 5-point scale from “very low” to “very high”, was dichotomized as “very low to moderate” and “high and very high”.

Warning signs were questioned in several ways. “Does <Name of the child> have (other) warning signs?” which can be answered with “no” or “yes, touch sensibility”, “yes, shrill screaming like I have never heard it before”, “yes, acting differently, clouded consciousness, apathy”, and “yes, seems seriously sick”. Specific warning signs like diarrhea/vomiting were dichotomized as having “no diarrhea or vomiting” and “having diarrhea and/or vomiting”. Rash was dichotomized as “no rash” and “yes, the redness can be pushed away or cannot be pushed away”. The parameter “dehydration” was based on the question “Are there signs of dehydration?—With the answer options “no”, “yes, dry mucous membranes”, “yes, the skin is dry and loose”, “yes, child looks very tired”, “yes, the eye-sockets appear sunken”, “yes, significantly fewer wet diapers than normal”, “yes, the fontanelle is sunken”.

Symptoms were also questioned in several ways, and the question “Does your child have symptoms?” could be answered with “no” or “cough”, “constrained breathing”, “freezing/chills”, “fatigue”, “sense of smell/taste disturbed”, “mucus”, “tonsillitis”, “joint swelling”, “teething”, and “other symptoms”.

Lastly, pain was operationalized with the question “Does your child have pain?—“no” or “yes, in the limbs”, “yes, in the head”, “yes, in the neck”, “yes, in the ear”, “yes, in the stomach”, and “yes, somewhere else”. Because the variable pain had a high number of missing values (*n* = 1369), it was recoded, with missing values defined as “no pain”. This is a plausible assumption because no entries were mandatory.

Antipyretics use is not questioned directly within the FeverApp, but was operationalized based on the answers parents gave regarding the use of medications. All medications which can be defined as antipyretics were summarized together to a new variable, “antipyretics use”, if parents gave no medication or a different one the variable was coded as “no antipyretics use”.

Parental confidence was operationalized with the question “When <Name of the child> has a fever, do you feel confident to deal with it appropriately?” and a 5-point Likert scale from thumb down to thumb up/red to green (Figure 1c). Because the dependent variable antipyretics was dichotomous, the skewed distributed parental confidence was also dichotomized as “low to moderate” and “high and very high” for a better comparison.

### 2.5. Statistical Analysis

IBM SPSS, Version 28 (IBM, Armonk, NY, USA) was used for data analysis. Logistic regression requirements were tested before and within regression analyses [22]. Metric independent variables were tested for linearity with the logit [23]. Children’s age did not show linearity with the logit, so it was converted into a dichotomous variable based on a median split.

Binary logistic regression was performed to test for univariate associations. Multivariate logistic regression analyses were performed to see how associations with the independent variables differed within a model. Based on the univariate analyses, significant independent variables were included in the multivariate analyses using the enter method.

## 3. Results

### 3.1. Descriptive Statistics

In this study, 3721 first entries on febrile children from the separate families (siblings were excluded) entry were analyzed. Parents average age was 34 years (SD = 4.95) and 51.1% of them had a higher school education, which is above the average in Germany [24]. The proportion of individuals with migration background (defined as living in Germany without German nationality) was 8.9%, which corresponds to two-thirds of the population with a migration background (defined by non-German citizenship) in Germany [25].

In general, parents reported greater confidence in managing their children’s fever (58%), and a small percentage of parents gave antipyretics (14.7%). Children’s age was right-skewed, distributed on average 21 months with a very high deviation (SD = 22.97). Due to a large number of younger children, the median is 12 months (IQR: 7 to 26 months) and the range covers the complete selection from 0 months to 17 years. The gender was approximately equally distributed with 47.4% female children.

Regarding medical background, almost half of the children had no episode of fever in the last 12 months (41.4%). The first recording included no acute fever measurement (threshold 38.5 °C) in almost half of the children (44.5%), but for most of the children, low to moderate well-being was reported (82.3%). General warning signs (e.g., touch sensitivity, shrill screaming) were reported less frequently (18.9%). Specific warning signs such as rash (4.8%), diarrhea (14.2%) and dehydration (15.2%) were even less common in the first recording. A high percentage of children (84.4%) showed symptoms (e.g., coughing, freezing/chills, constrained breathing) and nearly one-third of children had pain (e.g., pain in the limbs, in the neck, in the head) (28.2%) (Table 1).

### 3.2. Logistic Regression with the Dependent Variable Parental Confidence

#### 3.2.1. Bivariate Associations of Parental Confidence and Fever-Relevant Parameters

Higher parental age [OR = 1.014, 95% CI: 1.001–1.028; *p* = 0.041] was significantly associated with parental confidence but increased the likelihood of being confident by just 1%. Children older than 12 months [OR = 1.608, 95% CI: 1.405–1.839; *p* < 0.001], children with at least one episode of fever in the last 12 months [OR = 1.589, 95% CI: 1.377–1.833; *p* < 0.001] and children’s high well-being [OR = 1.693, 1.396–2.052; *p* < 0.001] were positively associated with being confident in managing fever in children and increased the likelihood up to 70%.

In contrast, having a female child decreased the likelihood of being very confident by about 25 % [OR = 0.746, 95% CI: 0.652–0.853; *p* < 0.001]. Warning signs [OR = 0.453, 95% CI: 0.380–0.539; *p* < 0.001] and dehydration [OR = 0.567, 95% CI: 0.470–0.685; *p* < 0.001] decreased the likelihood of high parental confidence by 50%. Presence of fever [OR = 0.699, 95% CI: 0.610–0.800; *p* < 0.001], diarrhea [OR = 0.783, 95% CI: 0.648–0.948; *p* = 0.012] and symptoms [OR = 0.694, 95% CI: 0.572–0.843; *p* < 0.001] negatively affected the likelihood of having high confidence by approximately 30% (Table 2).

#### 3.2.2. Multivariate Associations of Parental Confidence and Fever Relevant Parameters

Higher age group (>1 year) of the children [OR = 1.498, 95% CI: 1.244–1.803; *p* < 0.001] and if they had at least one episode of fever in the past 12 months [OR = 1.522, 95% CI: 1.271–1.822; *p* < 0.001) increased the likelihood of being highly confident each by about 50%. If the child was female [OR = 0.770, 95% CI: 0.655–0.906; *p* = 0.002], had fever [OR = 0.673. 95% CI: 0.566–0.800; *p* < 0.001] or showed signs of dehydration [OR = 0.646, 95% CI: 0.515–0.811; *p* < 0.001), the likelihood of being highly confident decreased by about 30%. When children showed general warning signs [OR = 0.494, 95% CI: 0.401–0.608; *p* < 0.001], the likelihood of being highly confident decreased by about 50%. The logistic regression model was statistically significant, χ²(10) = 179.476, *p* < 0.001, with a very small amount of explained variance shown by Nagelkerke’s *R*² = 0.089 [26]. See further results in Table 3.

### 3.3. Logistic Regression with the Dependent Variable Antipyretic Use

#### 3.3.1. Bivariate Associations of Antipyretics Use and Fever Relevant Parameters

Regarding the immediate use of antipyretics, occurrence of fever [OR = 3.477, 95% CI: 2.706–4.467; *p* < 0.001] had the strongest increasing effect on the likelihood of using antipyretics. If the children showed symptoms [OR = 2.191, 95% CI: 1.520–3.157; *p* < 0.001], had at least one episode of fever in the past 12 months [OR = 1.946, 95% CI: 1.524–2.484; *p* < 0.001], or showed current warning signs [OR = 1.878, 95% CI: 1.421–2.482; *p* < 0.001], the likelihood of using antipyretics doubled or nearly doubled. For the group of children older than 12 months [OR = 1.686, 95% CI: 1.346–2.112; *p* < 0.001], if children showed pain [OR = 1.652, 95% CI: 1.304–2.092; *p* < 0.001] or signs of dehydration [OR = 1.521, 95% CI: 1.132–2.043; *p* = 0.005], the likelihood of using antipyretics was increased by 50% to 70%.

In contrast, high parental confidence [OR = 0.753, 95% CI: 0.601–0.943; *p* = 0.013] and high well-being [OR = 0.215, 95% CI: 0.144–0.321; *p* < 0.001] decreased the likelihood of antipyretics use by about 25% to 80% (Table 4).

#### 3.3.2. Multivariate Associations of Antipyretics Use and Fever-Relevant Parameters

High child well-being decreased the likelihood of antipyretic use by approximately 60% [OR = 0.373, 95% CI: 0.220–0.632; *p* < 0.001], whereas high parental confidence decreased the likelihood of antipyretic use by approximately 30% [OR = 0.726, 95% CI: 0.550–0.960; *p* = 0.024].

Fever increased the likelihood of antipyretic use the most [OR = 2.576, 95% CI: 1.897–3.500; *p* < 0.001], followed by at least one episode of fever in the past 12 months [OR = 1.775, 95% CI: 1.300–2.425; *p* < 0.001] and warning signs [OR = 1.501, 95% CI: 1.078–2.090; *p* = 0.016]. The logistic regression model was statistically significant, χ²(9) = 135.390, *p* < 0.001, with a small amount of explained variance shown by Nagelkerke’s *R*² = 0.124 [26]. See detailed results in Table 5.

## 4. Discussion

### 4.1. Aim of the Study and Discussion of Descriptive Analyses Results

The aim of the study was to examine the associations of fever-related parameters at the beginning of a fever episode in relation to initial parental confidence or the immediate use of antipyretics. The purpose was to investigate whether parents’ confidence and behaviors during initial fever management were associated with the actual poor condition of the children or by an underlying general concern.

In general, most of the parents were very confident in managing their children’s fever (58%) and the percentage of antipyretics use was small (14.7%) in this sample. Other studies show a higher percentage of anxious parents [6,8], although a German study found a similar level of confidence among parents [12]. It should be noted that the former studies examined an emergency room, a current care visit or a health maintenance visit, whereas the German study examined the parents of healthy children. Given that the FeverApp data tend to be from an acute fever episode, it is noteworthy that the confidence level is so high.

Other studies have shown a higher percentage of antipyretics use [27,28,29] considering a complete fever episode. This can be explained by the following factors: First, the FeverApp contains guideline-based [30] text and video material which is demonstrably [31] effective in letting users know that antipyretics should primarily be used in case of pain or strongly impaired well-being and not solely with the aim of lowering body temperature. Second, only the first measurement of a fever episode was analyzed. Third, about one third of data regarding medication was missing.

Even though we did not find an association between migration background and school degree, these characteristics were observed to influence anxiety and use of antipyretics in other studies [11,32]. This may be explained since migration background is defined as “living in Germany with or without a German nationality”, whereas in other studies it is defined differently. Therefore, individuals with a migration background who also have a German citizenship are not included [11,25]. In addition, other studies also include higher education degrees and income, whereas in the current study, only the first school degree was considered, which might influence the results [11,32].

### 4.2. Discussion of Bivariate and Multivariate Logistic Analyses Results

In the bivariate analyses, the group of children older than 12 months and having at least one episode of fever in the past 12 months were associated with high parental confidence, increasing its likelihood by approximately 60%. In contrast to this result, Walsh et. al. [20] hypothesized that parental experience with a febrile child is negatively affected by parental confidence.

High well-being had the largest increasing effect (70%) on the likelihood of being very confident. Warning signs, signs of dehydration decreased the likelihood of high parental confidence by 40 to 50%, and symptoms and fever decreased the likelihood of high parental confidence by 30%. This may suggest that parents in this study were not only paying attention to their child’s temperature but were actually paying more attention to warning signs and well-being and were more likely to assess their child’s overall health.

The multivariate analyses regarding parental confidence showed that at least one febrile episode in the past 12 months, older age group of children, warning signs, current fever, dehydration and gender of children had significant associations with parental confidence. It seems plausible that previous experience with a febrile child, which implicates the older age group, most strongly increases the likelihood of confidence because it showed parents that they can manage the fever. Children’s age below one year should be taken more seriously [14]. Warnings signs, such as dehydration and current febrile temperature should be taken into account together, according to the analyzed model.

Regarding the use of antipyretics, elevated temperature had the strongest increasing influence on the likelihood of using antipyretics in the bivariate analyses, although it is not necessarily recommended to administer antipyretics when fever is low [14,15] but rather to comfort the child when it is in pain, has symptoms or shows warning signs [15]. Symptoms, warning signs and high level of well-being doubled or almost doubled the likelihood of using antipyretics, which thus shows that fever does indeed have a strong association, but other factors are also considered.

At least one fever episode in the past 12 months, as an indicator of parent’s experience with a febrile child, increased the likelihood of being confident, but also the use of antipyretics by about 70%. The results could be an indicator of an effect of antipyretics on parental confidence. Because parents had previous experience with a febrile child, they might have used antipyretics to reduce their anxiety and their child’s symptoms, so they felt more confident as a result of the self-efficacy via administration of antipyretics.

Parental confidence decreased the likelihood of using antipyretics by 30%. This shows that parental confidence does indeed have a significant association with the parental management of fever, but the actual poor condition of the child is still more important than parental confidence or anxiety.

The multivariate model regarding the use of antipyretics showed that current fever, at least one febrile episode in the past 12 months, warning signs, high well-being and parental confidence had significant associations with the use of antipyretics in children. Parental confidence was associated with a 30% lower likelihood of antipyretic use, but fever and high well-being had the highest association with antipyretic use in bivariate and multivariate analyses.

In general, the models showed that parents do not over interpret the child’s temperature or any underlying anxiety, but also pay attention to well-being, warning signs, dehydration, and their own experience and the children’s age. This is in accordance with the literature, which recommends that more attention be paid to children’s general well-being than just temperature in fever management [15], although in practice, this is sometimes different [3,4,5,6,8,9,13,16].

### 4.3. Strengths and Limitations of the Study

The FeverApp registry is an app-based tool and an ecological momentary assessment for recording current fever episodes so that naturalistic associations can be made. This can be considered a major strength of the data. Actual behavior is being recorded and not only attitudes or memories of behavior. Since we conducted a logistic regression within a model, the different correlations of several parameters can be mapped in relation to each other. Another part to add is the high number of children we could analyze.

However, there are some limitations of the study which have to be addressed. The FeverApp is designed as a documentational tool to observe guideline adherence. It also serves as an informational tool since informations about the observed guidelines have to be provided. This may lead to a bias in the interpretation of the construct parental confidence and the management of their children’s fever. Our goal was to do a cross-sectional observation of parent’s confidence and fever relevant data, not to investigate an intervention. But parents may already be influenced by the FeverApp and feel less anxious and better informed when it comes to managing their children’s fever with the information provided within the app. Even if the first record entry was selected, parents may already feel more confident in having such a tool. In addition, users are presented with an informational video after installing the app, which could also have an impact. The video showed significant changes in some outcomes related to fever management in one study [31], so it may have already affected parental confidence and recommended management. On the other hand, Schwarz et al. [33] examined the interactions of users of the FeverApp and showed that the main usage of the FeverApp is for documentation, not information.

A further limitation is the cross-sectional perspective itself because this analysis consider solely the first entry. Therefore, causality could not be drawn. Because parental experience was positively associated with parental confidence and use of antipyretics, this could be an indicator that antipyretics were used to increase confidence. This would have been a different causal relationship than assumed, and relates to an illusion on the side of the parents as antipyretics do not increase safety.

Looking at the first entry is another limitation, since we did not look at a whole febrile episode but rather the beginning of it, so the results might be different from a different time point. Because logistic regression was used, the variables were dichotomized, which was a loss of information and may have confounded the results.

Due to the naturalistic data collection, missing values could not have been excluded. It has to be considered that antipyretics had a lot of missing values and it is not clear whether parents did not give antipyretics or forgot to record it, even though it was not proven that the missing values were systematic and not random in subgroups. Nevertheless, the correlations within the logistic model of antipyretic use have to be interpreted with caution.

Both multivariate models have a low explained variance for the dependent variable. Therefore, the generalizability of the results is questionable.

Lastly, parental confidence was questioned with only one question and might not operationalize the construct correctly. Other studies mainly discussed fear, not confidence. We tried to approach this study in a more salutogenetic way of questioning parents feelings, but comparisons with other studies are now harder to make.

### 4.4. Implications for Future Research

The study population was not very diverse and could not represent the German population since there were fewer people with a migration background and the majority had a high educational status. It would be interesting for future research to observe a sample with a more diverse socioeconomic status and a higher percentage of people with a migration background.

Since the explained variance of models was low, further models could examine other variables or a combination of variables; for example, if parents had contact with their doctor or what other measures were taken apart from antipyretics. Another time point within a fever episode, for example, with the highest reported temperature, would be a good addition.

## 5. Conclusions

Parents in this study showed mostly high confidence in managing their children’s fever in the first entry; parameters associated with parental confidence showed that they had a reasonable sense of confidence based on a combination of relevant fever parameters rather than a single major factor. Use of antipyretics was low in the first entry of this population, and the parameters associated with them were partly in line with recommendations. However, febrile temperature still had the highest association with the use of antipyretics. This indicated that further improvements in the level of parental education on fever is needed. Since there are some promising results in this sample regarding parental fever management in line with recommendations, further research could be conducted to examine the impact of the FeverApp on parental confidence, antipyretics use and other parameters.

## Figures and Tables

**Table 1:** Descriptive statistics of parents’ and children’s sociodemographic and fever-related parameters.

Parents		*n*	Missings
Age in years ^1^ (M, SD)	33.96 (4.94)	3721	0
School degree ^1^ (%, *n*)			
General qualification for university entrance	51.1 (1903)	3648	73
Advanced technical college entrance qualification	21.0 (783)		
Intermediate school-leaving certificate	20.7 (771)		
Lower secondary school-leaving certificate	4.6 (173)		
none	0.5 (18)		
Migration background ^1^ (%, *n*)	8.9 (330)	3721	0
Confidence in children’s fever management ^2^ (%, *n*)		3530	191
very low–moderate	39.8 (1481)		
high–very high	58.0 (2049)		
**Children**			
Female ^1^ (%, *n*)	47.5 (1767)	3721	0
Age in months ^1^ (M, SD)	20.92 (22.97)	3721	0
Fever episode in the last 12 months ^1^ (%, *n*)		3355	366
Yes	58.6 (1967)		
No	41.4 (1388)		
Temperature ^2^ (%, *n*)		3721	0
under 38.5 °C	44.5 (1656)		
38.5 °C and over	55.5 (2065)		
Well-Being ^2^ (%, *n*)		3343	378
very low–moderate	82.3 (2751)		
high–very high	17.7 (592)		
Warning signs ^2^ (%, *n*)	18.9 (644)	3405	316
Rash ^2^ (%, *n*)	4.8 (165)	3465	256
Diarrhea ^2^ (%, *n*)	14.2 (508)	3574	147
Dehydration ^2^ (%, *n*)	15.2 (527)	3461	260
Symptoms ^2^ (%, *n*)	84.4 (2966)	3514	207
Pain ^2^ (%, *n*)	44.6 (1050)	2352	1369
Pain imputed ^2^ (%, *n*)	28.2 (1050)	3721	0
Antipyretics dose ^2^ (%, *n*)		2513	1208
No	85.3 (2144)		
Yes	14.7 (369)		

Notes: ¹ stable variable; ² current, initial state during a fever episode.

**Table 2:** Frequencies and associations of parental confidence regarding children’s management of fever.

	Parental Confidence				
	Low	High	*n*	OR [95% CI]	*p*-Value
Parents					
Age in years (M, SD)	33.76 (4.848)	34.11 (5.023)	3530	1.014 [1.001–1.028]	0.041
Higher school degree (%, *n*)	53.4 (774)	51.1 (1029)	3463	0.913 [0.798–1.046]	0.189
Migration background (%, *n*)	9.5 (140)	8.3 (171)	3530	0.872 [0.690–1.102]	0.252
Children					
Female (%, *n*)	51.5 (763)	44.2 (906)	3530	0.746 [0.652–0.853]	<0.001
Age 12 months and over (%, *n*)	46.4 (687)	58.2 (1192)	3530	1.608 [1.405–1.839]	<0.001
At least one fever episode in the last 12 months (%, *n*)	52.3 (699)	63.5 (1175)	3187	1.589 [1.377–1.833]	<0.001
Fever (%, *n*)	60.9 (902)	52.1 (1068)	3530	0.699 [0.610–0.800]	<0.001
High well-being (%, *n*)	13.3 (181)	20.6 (384)	3226	1.693 [1.396–2.052]	<0.001
Warning signs (%, *n*)	26.3 (360)	13.9 (274)	3340	0.453 [0.380–0.539]	<0.001
Rash (%, *n*)	5.1 (72)	4.5 (89)	3384	0.875 [0.636–1.203]	0.412
Diarrhea/Vomiting (%, *n*)	16.1 (234)	13.1 (265)	3485	0.783 [0.648–0.948]	0.012
Dehydration (%, *n*)	19.8 (275)	12.3 (244)	3373	0.567 [0.470–0.685]	<0.001
Symptoms (%, *n*)	87.3 (1252)	82.7 (1662)	3444	0.694 [0.572–0.843]	<0.001
Pain imputed (%, *n*)	28.9 (428)	28.7 (589)	3530	0.993 [0.856–1.150]	0.921

**Table 3:** Results of multivariate logistic regression analyses of the dependent variable parental confidence.

Independent Variables	OR	95% CI	*p*-Value
Constant	2.389		<0.001
Parents			
Age in years	0.992	[0.976–1.009]	0.382
Children			
Female	0.770	[0.655–0.906]	0.002
Age in Months 12 or older	1.498	[1.244–1.803]	<0.001
At least one fever episode in the last 12 Months	1.522	[1.271–1.822]	<0.001
Fever	0.673	[0.566–0.800]	<0.001
High well-being	1.252	[0.993–1.580]	0.058
Warning signs	0.494	[0.401–0.608]	<0.001
Diarrhea/Vomiting	1.055	[0.837–1.330]	0.649
Dehydration	0.646	[0.515–0.811]	<0.001
Symptoms	0.814	[0.640–1.035]	0.093

Notes: Nagelkerke’s *R*^2^ = 0.089; Model χ²(10) = 179.476 (*n* = 2629; *p* < 0.001).

**Table 4:** Frequencies and associations of antipyretics use regarding children’s management of fever.

	Use of Antipyretics				
	No	Yes	*n*	OR [95% CI]	*p*-Value
Parents					
Age in years (M, SD)	33.88 (4.928)	33.91 (4.555)	2513	1.001 [0.979–1.024]	0.923
Higher school degree (%, *n*)	54.6 (1154)	51.3 (184)	2474	0.876 [0.700–1.095]	0.245
Migration background (%, *n*)	8.0 (172)	8.7 (32)	2513	1.089 [0.734–1.615]	0.673
High confidence (%, *n*)	61.4 (1291)	54.4 (196)	2464	0.753 [0.601–0.943]	0.013
Children					
Female (%, *n*)	47.8 (1024)	50.1 (185)	2513	1.100 [0.882–1.372]	0.399
Age 12 months and over (%, *n*)	47.5 (1019)	60.4 (223)	2513	1.686 [1.346–2.112]	<0.001
At least one fever episode in the last 12 months (%, *n*)	52.4 (1002)	68.1 (233)	2256	1.946 [1.524–2.484]	<0.001
Fever (%, *n*)	46.4 (995)	75.1 (277)	2513	3.477 [2.706–4.467]	<0.001
High well-being (%, *n*)	22.5 (441)	6.4 (21)	2289	0.234 [0.148–0.369]	<0.001
Warning signs (%, *n*)	14.4 (295)	24.0 (81)	2383	1.878 [1.421–2.482]	<0.001
Rash	4.7 (96)	5.1 (18)	2400	1.100 [0.656–1.843]	0.719
Diarrhea/Vomiting	12.0 (254)	14.5 (52)	2474	1.241 [0.900–1.712]	0.189
Dehydration	13.6 (278)	19.3 (67)	2392	1.521 [1.132–2.043]	0.005
Symptoms (%, *n*)	80.8 (1685)	90.2 (323)	2443	2.191 [1.520–3.157]	<0.001
Pain imputed (%, *n*)	24.1 (517)	34.4 (127)	2513	1.652 [1.304–2.092]	<0.001

**Table 5:** Results of multivariate logistic regression analyses of the dependent variable use of antipyretics.

Independent Variables	OR	95% CI	*p*-Value
Constant	0.063		<0.001
Parents			
High confidence	0.726	[0.550–0960]	0.024
Children			
Age in Months 12 or older	1.015	[0.752–1.371]	0.920
At least one fever episode in the last 12 Months	1.775	[1.300–2.425]	<0.001
Fever	2.576	[1.897–3.500]	<0.001
High well-being	0.373	[0.220–0.632]	<0.001
Warning signs	1.501	[1.078–2.090]	0.016
Dehydration	0.880	[0.606–1.278]	0.502
Symptoms	1.308	[0.835–2.048]	0.241
Pain imputed	1.235	[0.922–1.654]	0.157

Notes: Nagelkerke’s *R*² = 0.124; Model χ²(9) = 135.390 (*n* = 1847; *p* < 0.001).

## Data Availability

The datasets analyzed during the current study are available from the corresponding author on reasonable request.
